# Magnitude and determinants of change in objectively-measured physical activity, sedentary time and sleep duration from ages 15 to 17.5y in UK adolescents: the ROOTS study

**DOI:** 10.1186/s12966-015-0222-4

**Published:** 2015-05-14

**Authors:** Paul J Collings, Katrien Wijndaele, Kirsten Corder, Kate Westgate, Charlotte L Ridgway, Stephen J Sharp, Valerie Dunn, Ian Goodyer, Ulf Ekelund, Soren Brage

**Affiliations:** Institute of Metabolic Science, MRC Epidemiology Unit, University of Cambridge, Box 285, Addenbrookes Hospital, Cambridge, CB2 0QQ UK; Developmental Lifecourse Research Group, Department of Psychiatry, University of Cambridge, Cambridge, UK; Department of Sport Medicine, Norwegian School of Sports Science, Oslo, Norway

**Keywords:** Motor activity, Energy Expenditure, Physical activity intensity, Sedentary time, Sleep duration, Reverse causation, Adolescents

## Abstract

**Background:**

Self-reported physical activity (PA) and sleep duration (SLP) change markedly throughout adolescence. We sought to quantify changes in objectively-measured PA, sedentary time (ST) and SLP through adolescence, and to investigate baseline body composition and baseline activity levels as determinants of change.

**Methods:**

Individually calibrated combined heart rate and movement sensing was used to estimate PA energy expenditure (PAEE), SLP, daily ST and time in light (LPA), moderate (MPA), vigorous (VPA), and moderate-to-vigorous physical activity (MVPA) in 144 adolescents (50 % boys) of mean age 15.1(±0.3)y at baseline and 17.5(±0.3)y at follow-up. Changes in PA (ΔPA), ST (ΔST) and SLP (ΔSLP) were calculated as follow-up minus baseline values. Waist circumference (WC) was measured at baseline and follow-up, as was fat mass index (FMI) and fat-free mass index (FFMI) by a pooled estimation method including bio-impedance. Comparison of baseline and follow-up activity was made by mixed-model ANOVA. Linear regression adjusted for baseline demographics, total and weekend hours of monitor wear time and the seasons of activity measurements, was used to investigate baseline body composition as determinants of ΔPA, ΔST and ΔSLP. A further model adjusted for baseline of the outcome assessed baseline activity as a predictor of behaviour change, and investigated associations for baseline body composition independent of the baseline level of the outcome.

**Results:**

From baseline to follow-up levels of MPA and VPA declined (*p* ≤ 0.039). The annual decline in MVPA was equivalent to -4.5 and -3.0 min/d in boys and girls, respectively. Baseline FMI, FFMI and WC were positively associated with ΔLPA and negatively associated with ΔST in boys when adjusted for baseline of the outcome (*p ≤* 0.037 for all). SLP increased from baseline to follow-up (*p* = 0.004) but ΔSLP was not associated with baseline body composition (*p* ≥ 0.13). For all variables, higher baseline levels were associated with greater declines over time (*p* ≤ 0.003).

**Conclusions:**

Levels of higher-intensity PA decline from mid-to-late adolescence, whereas the duration of sleep increases. Changes in LPA and ST may be associated with baseline body composition, but the baseline level of the outcome is consistently the strongest predictor of changes in adolescent activity.

## Introduction

Physical activity (PA) levels are thought to decline markedly throughout adolescence (10-19 years) [1]. The magnitude of this decline may be related to the baseline level of activity, with initially more active adolescents experiencing greater declines over time [1]. If true, this could have considerable public health implications. Increased levels of sedentary time (ST) and reduced sleep duration (SLP) are additional negative behavioural alterations that may occur through adolescence [2–5]. However, it is problematic that most studies to date have measured these behaviours by self-report [1]. In particular, self-or parent-reported methods are continually and commonly implemented for habitual SLP ascertainment in youth, but a vast array of tools exist and rarely have they been psychometrically tested to any extent [6]. The small amount of existing validity data reveal that childhood SLP is consistently over-reported [7], and that the accuracy of estimates is influenced by factors such as age [8] and weight status [9].

Single-piece body worn sensors which are lightweight and entail low participant burden have recently emerged as valuable tools for simultaneous measurement of habitual PA, ST and SLP. These devices offer high-resolution and time-stamped data which can be used to provide accurate estimates of the time engaged in disparate PA intensities [10, 11]. Even so, to date the few studies that have used these objective methods in children have tended to focus on changes in moderate-to-vigorous PA (MVPA) only [12]. This may mask important changes within this category, however, as it is becoming generally accepted that vigorous physical activity (VPA) confers health benefits over and above that of moderate physical activity (MPA). As such, the UK recommendation currently states that VPA should be performed on at least three days per week by 5-18 year olds [13]. Furthermore, light physical activity (LPA) contributes substantially to physical activity energy expenditure (PAEE) [14], and has been shown by some to exhibit negative associations with adiposity [15–17] and positive associations with various cardiometabolic biomarkers in childhood [18]. For these reasons, it is recommended that constrained focus on MVPA as the dominant health-related aspect of human movement should be stemmed, and that the entire range of PA intensities should be investigated [19], inclusive of ST which may be independently associated with adiposity [20] and CVD risk factors [21]. Sleep is the remaining activity-related behaviour that comprises 24 h of daily living. Adequate SLP is important for normal growth, development and functioning in youth [22–24] and is inversely associated with adiposity [25] and metabolic syndrome [26].

To our knowledge, no investigation has thus far quantified objectively and within a single sample changes in total and intensity-specific PA, ST and SLP through adolescence. Furthermore, unlike in younger children [27–32] and adults [33, 34], and despite consistent cross-sectional associations between these variables [20, 35], there is currently little evidence for baseline adiposity acting as a determinant of the changes in PA, ST or SLP between 10-19 years of age [36–39]. This is despite adolescents experiencing marked changes in activity behaviours, which tend to be most pronounced post-puberty when adiposity levels have risen sharply [40]. More studies are needed to identify the determinants of adverse changes in activity-related behaviours from mid-to-late adolescence, particularly considering the close proximity of this period to early adulthood, accompanied by evidence that childhood behaviours track to adult life [41]. This study intends to quantify changes in objectively-measured total and intensity-specific PA, ST and SLP from mid-to-late adolescence, and to investigate baseline body composition and baseline level of the respective PA, ST or SLP outcome as determinants of these changes.

## Methods

This study involves a random sub-sample of Cambridgeshire adolescents participating in the ROOTS study [42]. Initially, 1238 students were consented to the study, of which 1203 students attended for anthropometrical measurements and provided demographic data at wave 0 (mean (±SD) age: 14.5 ± 0.3y). Subsequently, 930 and 844 students were followed-up at waves 1 (15.0 ± 0.3y, hereon referred to as baseline) and 2 (17.5 ± 0.3y, hereon referred to as follow-up), respectively. The random sub-sample constituted 212 adolescents who were recruited from eight schools and were approached for PA testing at baseline and follow-up. All procedures were explained to participants who could choose to decline any part of the study. The ROOTS study was approved by the Cambridge research ethics committee.

Participants wore a combined heart rate and movement sensor (Actiheart, CamNtech Ltd, Papworth, UK) for 4 consecutive days without interruption, recording data in 30-sec epochs. Heart rate was individually calibrated using sub-maximal step-tests at baseline [43]. PAEE (kJ/kg/day) was estimated by branched equation modelling [44] and further summarised as time spent at levels of standard metabolic equivalents of task (METs), categorised as: ST (≤1.5 METs exclusive of sleep), LPA (1.5-4 METs), MPA (4-7 METs), VPA (>7 METs), and MVPA (>4 METs). Participants were categorised at baseline and follow-up according to whether they adhered to the guideline daily volume of MVPA, by engaging in ≥60 min MVPA on average over the observation period (no minimum bout duration was specified). To differentiate between ST and SLP, participants reported the time that they usually got up and went to bed as phrased in the validated ‘Sleep Habits Survey for Adolescents’ [45], separately for school nights and weekends. This information about normal bedtime routine was superimposed upon the heart rate and movement data to provide a region of interest (ROI) for objective markers of sleep onset and termination. Using the ROI as a guide, a single reviewer blinded to all other participant data visually inspected all plots and used the following rules to detect sleep: 1) Sleep onset constitutes the beginning of sustained low movement registration accompanied by a steady decline in heart rate, 2) Sleep termination constitutes the commencement of movement subsequent to long periods of barren movement, together with an abrupt elevation in heart rate. The accumulated time spent in ≤1.5 METs between a designated sleep onset and termination period was deemed to be the sleep duration. The remainder of all time engaged in ≤1.5 METs was considered ST.

A valid monitor wear period constituted ≥48 hours of data (≥32 weekday and ≥16 weekend hours) with the stipulation that these hours included approximately balanced data from all quadrants of a 24 h day (≥12 h from morning (3 am–9 am), noon (9 am–3 pm), afternoon (3 pm–9 pm) and night (9 pm–3 am) time-periods). This quadrant-specific inclusion criteria was incorporated to safeguard against possible bias arising from specific parts of days being over-represented, and was supplemented by a diurnal bias minimisation procedure [46]. Detailed information about the PA data is available elsewhere [14].

Participants were measured during one of three school terms: summer (April-July), autumn (September-December) or spring (January-March). The season of measurements was not standardised between baseline and follow-up, and previously we have reported differences in PA and ST levels between autumn and spring school terms compared to the summer term [14]. In order to account for seasonal activity patterns a three-level indicator variable was created. Participants were categorised as being measured in spring/autumn at both time points (58.3 %), in summer at baseline and spring/autumn at follow-up (27.1 %), or in spring/autumn at baseline and summer at follow-up (14.6 %). With regards other covariates, information concerning gender, ethnicity, pubertal status, and area-level SES were collected at wave 0 (0.6 ± 0.14y before baseline). Area-level SES was based on postcodes while pubertal status was assessed by self-reported menarcheal status, tanner stages, and levels of salivary testosterone in boys [14].

Baseline and follow-up height (m), weight (kg), BMI (kg/m^2^) and body-tissue impedance (Ω; Tanita TBF-300 MA, Tokyo, Japan) were measured. Fat mass index (FMI, kg/m^2^) and fat-free mass index (FFMI, kg/m^2.5^) were subsequently predicted using a pooled estimation approach [47] based on the aggregate mean of seven published equations (estimates from the Tanita TBF-300 MA bioelectrical impedance analyser which are based on unknown equations were also pooled) [48–54]. Waist circumference (WC) was measured at the umbilical level during mid-expiration in duplicate (or triplicate if the first two measurements differed by >0.5 cm) to the nearest 0.1 cm, and the mean of measurements were taken forward for analyses. For descriptive purposes, BMI and WC *z*-scores were computed based on British reference data [55, 56].

Differences between baseline and follow-up anthropometry, body composition and crude levels of PA, ST and SLP were examined by mixed-model ANOVA, in boys, girls and overall, with a test for gender × wave interaction to decipher if differences between baseline and follow-up varied by boys and girls. To investigate differences in characteristics between the included sample and all participants with valid activity data at baseline only, chi-squared tests and sex-adjusted linear regression with robust standard errors (to account for clustering by school) were performed.

Linear regression models with robust standard errors (again to account for clustering by school) were used to investigate baseline determinants of change in PA, ST and SLP (ΔPA, ΔST and ΔSLP, respectively, baseline subtracted from follow-up values). Separate models were created for ΔPAEE, the four PA intensity categories, ΔST and ΔSLP. For each outcome, baseline FMI, FFMI and WC were individually assessed as determinants of change. Models were adjusted for gender, baseline age, ethnicity, area-level SES, follow-up duration, the total and weekend hours of monitor wear time and the seasons (school terms) of activity measurements at baseline and follow-up (Model 1). Pubertal status was not added as a covariate due to low variation (90 % of participants were pubertal at wave 0) and missing data for one participant. Models with WC as the main exposure were further adjusted for height. A separate model (Model 2), including all covariates in Model 1 as well as baseline of the outcome, was constructed to assess baseline activity as predictors of behaviour change and to investigate associations for body composition variables independent of the baseline level of the outcome [57]. Gender interactions with baseline body composition (Models 1 and 2) and baseline of the outcome (Model 2) were examined by *F*-tests. Statistical analyses were conducted in Stata/SE 13.1.

## Results

**Fig. 1:**
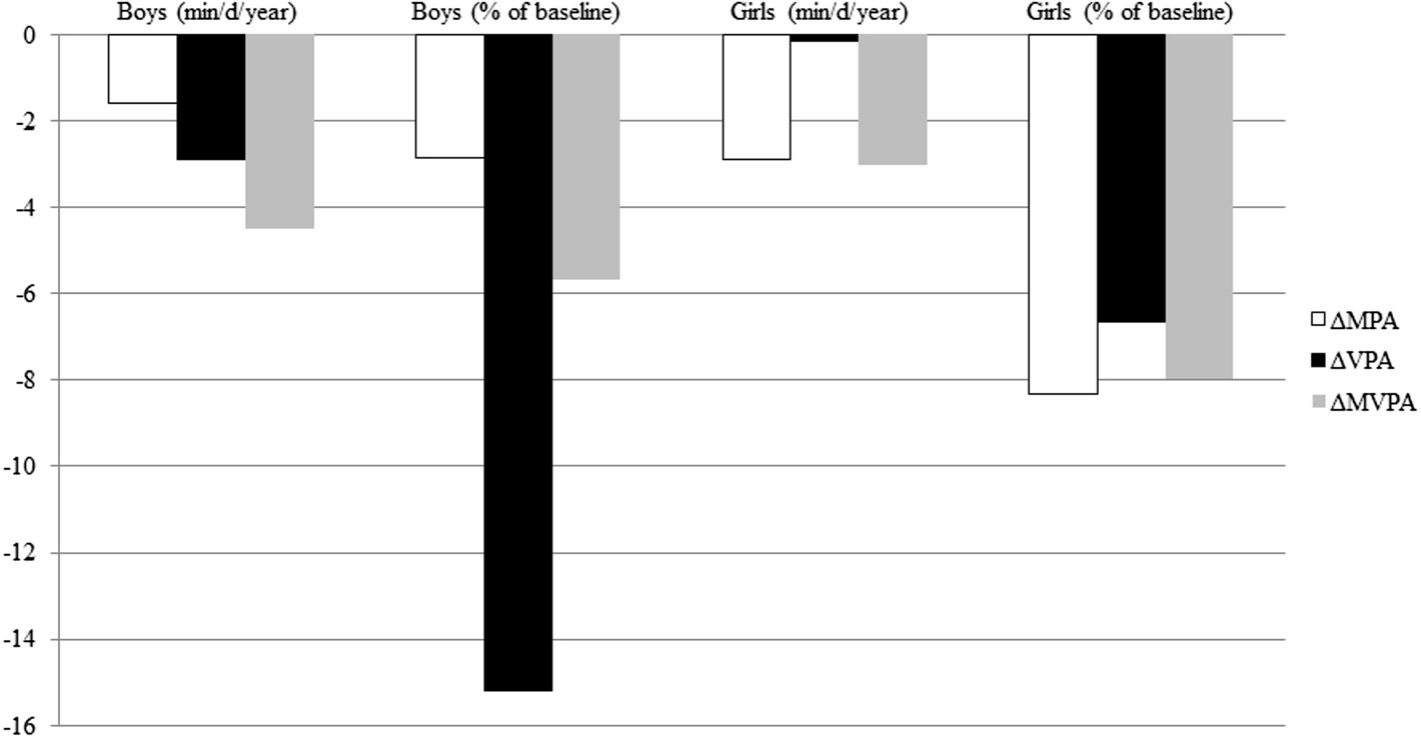
Mean annual changes in moderate, vigorous and moderate-to-vigorous physical activity, expressed in absolute units and as a proportion of the baseline level, stratified by gender

Of the 212 participants in the random sub-sample, 144 (67.9 %) provided valid activity data at baseline and follow-up and were included in analyses (Table 1). The sample was composed of 50 % boys, which was slightly different to the gender distribution of the 592 participants that possessed valid activity data at baseline only (41.6 % boys, *p* = 0.067). However, there were no differences in any other parameters between groups (*p* ≥ 0.10), including the levels of PA, ST and SLP at 15y of age (*p* ≥ 0.15). Participants were predominantly white (95 %) and from middle-to-high SES locations (85 %).The mean follow-up duration was 2.4(±0.2)y, during which time participants increased in all anthropometric and body composition dimensions (*p* < 0.001 for all) with boys acquiring more fat-free mass and abdominal adiposity (WC) than girls (*p* ≤ 0.043 for gender × wave interactions). Levels of MPA and MVPA declined over follow-up (*p* ≤ 0.039 for both), as did levels of VPA (*p* = 0.0038) but more so in boys than girls (*p* = 0.0087 for the gender × wave interaction). From baseline to follow-up SLP increased in both boys and girls (*p* = 0.004). The mean annual change in MPA, VPA, MVPA, expressed both in absolute terms and as a proportion of the baseline level, stratified by sex, is displayed in Fig. 1. MVPA declined by 4.5 and 3.0 min/d/year in boys and girls, respectively. When expressed as a proportion of baseline, the largest observed change was for VPA in boys, which declined by 15 % of the baseline level annually. SLP increased by 4.0 and 9.7 min/night/year in boys and girls, respectively.

**Table 1:** Characteristics of participants with valid PA and sedentary time data at baseline and follow-up

	Boys (*n* = 72)		Girls (*n* = 72)			
	Baseline	Follow-up	Baseline	Follow-up	*P*-value (follow-up vs baseline, boys and girls combined)	*P*-value for gender × wave interaction
Age (y)	15.1 ± 0.3^*1*^	17.4 ± 0.3	15.1 ± 0.3	17.5 ± 0.3	-	-
Weight (kg)	58.5 ± 10.2	68.1 ± 13.0	55.7 ± 8.2	59.8 ± 8.5	<0.001	<0.001
Height (cm)	171.8 ± 7.9	177.7 ± 6.7	163.1 ± 6.0	165.1 ± 6.0	<0.001	<0.001
BMI (kg/m^2^)	19.3 (3.3)^*2*^	20.8 (3.5)	20.4 (4.1)	21.3 (3.7)	<0.001	0.013
BMI *z*-score^*3*^	−0.030 ± 1.0	0.058 ± 1.1	0.19 ± 1.1	0.17 ± 1.0	0.47	0.27
Fat mass (kg)	7.6 (4.3)	9.1 (5.2)	14.6 (6.4)	15.4 (7.2)	<0.001	0.27
Fat mass index (kg/m^2^)	2.6 (1.4)	3.0 (1.6)	5.4 (2.4)	5.6 (2.5)	<0.001	0.65
Fat-free mass (kg)	49.7 (9.0)	55.5 (8.4)	40.8 (5.6)	43.2 (4.8)	<0.001	<0.001
Fat-free mass index (kg/m^2.5^)	16.7 (1.8)	17.7 (1.9)	15.2 (1.6)	15.7 ± 1.4	<0.001	<0.001
Waist circ. (cm)^*3,4*^	74.3 (6.6)	77.5 (9.1)	74.0 (12.2)	74.9 (10.5)	<0.001	0.043
Waist circ. *z*-score^*3*^	0.81 ± 0.72	0.75 ± 1.0	1.6 ± 1.0	1.7 ± 1.1	0.70	0.18
PAEE (kJ/kg/d)	83.1 ± 20.9	80.9 ± 23.1	62.3 ± 18.5	60.0 ± 19.8	0.19	0.98
LPA (min/d)	542.0 ± 105.4	557.0 ± 114.2	502.2 ± 107.7	490.9 ± 125.4	0.83	0.12
MPA (min/d)^*5*^	55.9 (31.9)	50.6 (34.7)	34.8 (30.6)	27.0 (25.1)	0.039	0.53
VPA (min/d)^*5*^	19.1 (29.7)	12.9 (21.3)	2.1 (6.4)	1.7 (6.3)	0.0038	0.0087
MVPA (min/d)^*5*^	79.5 (53.5)	67.5 (54.5)	37.6 (37.0)	31.3 (32.9)	0.0074	0.62
ST (min/d)	338.3 ± 110.4	324.4 ± 129.0	407.8 ± 118.7	403.9 ± 123.4	0.34	0.59
SLP (min/night)	478.0 ± 45.7	487.2 ± 54.8	486.3 ± 50.2	508.7 ± 59.8	0.0046	0.23

^*1*^Mean ± SD (all such values); ^*2*^Median (IQR) for all variables with skewed distributions; ^*3*^Based on British growth reference data; ^*4*^Due to missing data based on 140 participants (69 boys and 71 girls); ^*5*^Baseline estimates differ from Collings et al. (2014) due to different summary statistics, here, to complement the normally distributed change variables, median and IQRs are preferred to the geometric mean*;* analyses performed using mixed-model ANOVA, there were few minor changes in results when the natural log of skewed variables were used as the dependent variable or when school clustering was accounted for by using multilevel regression; there were also no differences in *p*-values when the statistics for activity variables were adjusted for baseline age and FMI, ethnicity, area-level SES, follow-up duration, the total and weekend hours of monitor wear time and the seasons (school terms) of activity measurements at baseline and follow-up. LPA, light physical activity; MPA, moderate physical activity; MVPA, moderate-to-vigorous physical activity; PAEE: physical activity energy expenditure; SLP, sleep duration; ST, sedentary time; VPA, vigorous physical activity

Table 2 shows associations of baseline body composition with ΔPA, ΔST and ΔSLP. Results from an unadjusted model (data not shown) were consistent with Model 1. As demonstrated by the considerable overlap of confidence intervals, results were not substantively different between Models 1 and 2, but Model 2 provided statistically significant evidence that higher baseline FMI, FFMI and WC were all associated with increased LPA and decreased ST in boys (*p ≤* 0.037 for all, associations were stratified by gender because of significant gender × body composition interactions for LPA and ST). Model 2 also provided some indication of an inverse association between baseline FMI and ΔVPA, although this was not statistically significant (*p* = 0.069).

**Table 2:** Associations between baseline body fat level and baseline level of the outcome with changes in PA/sedentary time (*n* = 144; 50 % boys; data are β (95 % CI))

	PAEE	*P*	ST	*P*	LPA	*P*	MPA	*P*	VPA	P	MVPA	P	SLP	P
	(Δ kJ/kg/day/year)		(Δ min/d/year)										(Δ min/night/year)	
FMI (kg/m^2^)^*1*^														
Model 1	0.17 (-0.49 to 0.82)	0.56	B:-6.0 (-16.1 to 4.1)	0.20	B: 4.8 (-1.3 to 10.8)	0.11	0.030 (-1.0 to 1.1)	0.95	0.0097 (-0.42 to 0.44)	0.96	0.040 (-1.0 to 1.1)	0.93	0.91 (-2.7 to 4.6)	0.57
			G:-0.098 (-7.2 to 7.0)	0.98	G:-0.54 (-6.9 to 5.8)	0.85								
Model 2	0.094 (-0.47 to 0.66)	0.71	B:-7.0 (-13.4 to -0.56)	0.037	B: 7.8 (5.6 to 10.0)	<0.001	-0.10 (-1.0 to 0.78)	0.79	-0.35 (-0.73 to 0.035)	0.069	-0.45 (-1.4 to 0.54)	0.32	-1.0 (-3.2 to 1.2)	0.31
			G:-0.17 (-6.8 to 6.4)	0.95	G:-0.51 (-5.0 to 6.1)	0.84								
FFMI (kg/m^2^)^*1*^														
Model 1	0.34 (-0.51 to 1.2)	0.38	B:-3.6 (-11.5 to 4.3)	0.32	B: 2.8 (-3.1 to 8.8)	0.30	0.16 (-1.3 to 1.6)	0.79	0.089 (-0.39 to 0.57)	0.68	0.25 (-1.2 to 1.7)	0.70	0.68 (-2.7 to 4.0)	0.65
			G: -1.1 (-12.7 to 10.5)	0.83	G: 0.076 (-7.8 to 9.3)	0.84								
Model 2	0.32 (-0.55 to 1.2)	0.41	B:-4.8 (-8.9 to -0.77)	0.026	B: 5.8 (4.2 to 7.4)	<0.001	0.13 (-0.31 to 0.57)	0.50	-0.26 (-0.80 to 0.29)	0.31	-0.12 (-0.78 to 0.54)	0.68	-1.6 (-3.8 to 0.57)	0.13
			G: 0.59 (-12.3 to 11.1)	0.91	G: 1.8 (-7.2 to 10.8)	0.65								
WC (cm)^*1,2*^														
Model 1	-0.0012 (-0.11 to 0.11)	0.98	B:-1.4 (-3.3 to 0.67)	0.16	B: 1.2 (0.17 to 2.3)	0.028	0.034 (-0.24 to 0.30)	0.78	0.0061 (-0.15 to 0.16)	0.93	0.040 (-0.27 to 0.35)	0.77	0.11 (-0.73 to 0.95)	0.77
			G: 0.71 (-0.50 to 1.9)	0.21	G:-0.86 (-1.9 to 0.23)	0.11								
Model 2	0.0092 (-0.092 to 0.11)	0.84	B:-1.7 (-3.1 to -0.34)	0.021	B: 1.8 (1.1 to 2.4)	<0.001	0.030 (-0.16 to 0.22)	0.72	-0.059 (-0.15 to 0.027)	0.15	-0.028 (-0.20 to 0.14)	0.71	-0.13 (-0.76 to 0.51)	0.66
			G: 0.51 (-0.77 to 1.8)	0.38	G:-0.54 (-1.5 to 0.46)	0.25								
Baseline of outcome	-0.20 (-0.23 to -0.17)	<0.001	-0.15 (-0.23 to -0.073)	0.003	-0.15 (-0.23 to -0.072)	0.003	-0.30 (-0.35 to -0.24)	<0.001	-0.32 (-0.42 to -0.22)	<0.001	-0.31 (-0.36 to -0.26)	<0.001	-0.33 (-0.39 to -0.27)	<0.001

Statistical comparisons made by linear regression accounting for within-school clustering; Model 1: adjusted for baseline age, sex, area-level SES, ethnicity, follow-up duration, the total and weekend hours of monitor wear time and the seasons (school terms) of activity measurements at baseline and follow-up; WC models were additionally adjusted for baseline height; Model 2: as per model 1 but also including baseline value of the outcome. Coefficients represent the expected mean change (95 % confidence interval in parentheses) in PA, ST or SLP per unit increase in independent variables. LPA, light physical activity; MPA, moderate physical activity; MVPA, moderate-to-vigorous physical activity; PAEE: physical activity energy expenditure; SLP, sleep duration; ST, sedentary time; VPA, vigorous physical activity. ^*1*^Results for ST and LPA are stratified by gender (B, Boys; G, Girls) because of significant sex*fatness interactions, for all other variables results are for genders combined; ^*2*^Due to missing data based on 140 participants (69 boys and 71 girls)

Associations between baseline levels of PA, ST and SLP and changes in these variables over follow-up are also shown in Table 2; there were no significant gender interactions (*p* ≥ 0.25 for all). Without exception, higher values of baseline behaviours were predictive of greater declines in those behaviours (*p ≤* 0.003 for all), and the size of associations were greatest for SLP and higher-intensity PA. Every additional min/night of baseline SLP and min/d of baseline MPA or VPA was associated with an approximate 0.3 units greater annual decline in these variables over follow-up; this was only 0.15 min/d for LPA and ST. With regards adherence to the guideline amount of daily MVPA, 68 participants engaged in ≥60 min MVPA/d at baseline and 57 % of these ‘sufficiently active’ participants continued to be active enough at follow-up. Of the 76 participants that were ‘insufficiently active’ at baseline, 80 % persevered to engage in <60 min MVPA/d at follow-up, with 48 % of boys and 93 % of girls persistently failing to meet guidelines.

## Discussion

In the ROOTS study we have previously shown relatively low levels of MPA and VPA performed by 15y old adolescents [14], which is in line with data from the Health Survey for England [58]. This study extends these observations, and shows that the annual change in MVPA from 15 to 17.5y of age in Cambridgeshire adolescents is equivalent to -4.5 and -3.0 min/d in boys and girls, respectively. The results are similar to those of Corder et al. [59], who reported that MVPA decreased by 3.9 and 2.0 min/d per year from the ages of 10-14, in boys and girls from a comparable location to the current study. However, they are considerably different to Nader et al. [12], who observed annual MVPA decreases of approximately 40 min/d in American children from 9-15y of age, although this change may have been exaggerated by the use of age-specific prediction equations [60]. Besides different study locations and different methods to assess PA, the differences in results could also be explained by dissimilar age and puberty statuses. The sample of Nader et al. [12] spanned the pubertal timeframe, which may have exerted a substantial negative effect on activity levels [40].

An important feature of this investigation is that variables other than MVPA were studied. While PAEE, LPA, and ST did not change over time in either gender, levels of MPA and VPA were lower at follow-up. VPA declined modestly in girls likely because their initial participation in VPA was very low, whereas in boys the mean annual rate of decline in VPA (-2.9 min/d/year) was the greatest of any observed changes when expressed as a proportion of baseline (-15 %). The same was also found by Corder et al. [59], and is seemingly important as VPA may confer health benefits beyond that of other PA intensities, such as lower adiposity [61] and elevated cardiorespiratory fitness, muscle and bone strength [13, 62]. Others have reported increased ST with advancing age in childhood, by a factor of about 30 min/d annually [2, 59], but the current study is unique as it involved uninterrupted combined accelerometry and heart rate monitoring, with separation of ST and sleep on the basis of self-reported bedtimes and reviewer identification of objective sleep markers [14]. We observed that SLP, not ST, increased from mid-to-late adolescence by 6.4 ± 30.0 min/night/year in boys and girls combined. This is contradictory to changes in self-reported SLP, which generally show that SLP diminishes across the adolescent age range by approximately 7.5-11 min/night/year [3–5]. Interestingly, a review of 18 small-scale studies conducted between 1972 and 2003 established that childhood SLP (typically ascertained by laboratory polysomnography or actigraphy) decreased with age only when recordings were made on school days, whereas on non-school days the duration of sleep remained unchanged from childhood to the end of adolescence [63]. This may go some way to explain our distinctive finding, as in this particular study weekday and weekend SLP were essentially given equal weighting due to our 4-day measurement protocol, which included an entire weekend. Nonetheless, our observation of lengthened SLP from mid-to-late adolescence should be construed as promising. Prevalence estimates of fatigue and feeling ‘worn out’ have markedly increased in adolescents since the mid-1980s [64]. Furthermore, we recently reported from the ROOTS study that inverse prospective associations exist between SLP and FMI gain in adolescent boys [25].

With regards the determinants of activity change, associations between higher adiposity with declining levels of accelerometer-measured total activity and MVPA have been observed in younger children with the same initial level of activity [27, 28, 31, 32, 65], but the same has not yet been reported in adolescents [36–39]. Furthermore, mixed results are available for the association between adiposity and ΔST [37, 39, 65], and we are aware of only one study in adults that has investigated adiposity as a predictor of ΔSLP [34]. When investigating the chronology of associations between activity and adiposity it is important to consider that differences in how precisely these constructs are measured can complicate inferences about the direction of causality [66]. In this respect, we advantageously report results that are based on combined heart rate and movement sensing. We have further reported results that are both unadjusted (Model 1) and adjusted (Model 2) for the baseline level of activity [67]. We found broad agreement in point estimates and confidence intervals between models, although they pose two subtly different questions and for this reason we would not necessarily expect the same conclusions [68]. Model 1 attempts to assess whether baseline body composition is associated with change in behaviour in individuals, regardless of their initial activity level, whereas Model 2, the favoured approach by most researchers, assesses whether baseline body composition is associated with change in activity for individuals with the same initial activity level. Adjusting for the baseline value of the outcome has been advocated in randomised clinical trials as a way of increasing efficiency where the randomised groups are comparable at baseline; however, in observational studies the distribution of the outcome at baseline may not be the same in individuals with similar levels of the exposure [68]. It has been suggested that in some situations adjusting for baseline does not answer a meaningful question [68], or doing so may produce a biased estimate of the association between exposure and change in outcome [69, 70]. In spite of these concerns, and reassuringly for the established literature which has relied on baseline adjusted results, we found general consistency between Models 1 and 2.

Model 2 in particular provided evidence that baseline FMI, FFMI and WC were all positively associated with ΔLPA and negatively so with ΔST in boys. These results likely reflect larger boys substituting some ST for LPA over follow-up, as the associations between body composition and ΔST were similar in magnitude, but opposite in direction, to the associations for ΔLPA. This switching between ST and LPA by larger boys may be perceived to reflect a saturation point for ST. That is, larger boys may have been so sedentary at baseline that they could only feasibly have decreased in this behaviour over follow-up, by substituting some ST for LPA. However, we have no strong evidence for this, particularly as the mean ST in adolescents at baseline was not overwhelming, and because no differences were found in the level of ST performed by normal and over fat ROOTS participants at 15y of age (baseline) [14]. The largest divergence of results between Models 1 and 2 was for FMI in relation to VPA, as significance levels changed considerably and the sign of effect changed direction when adjusting for baseline of the outcome. This may be explained by individuals with high FMI at baseline also having lower initial levels of VPA (*r* = -0.42). Without adjustment for baseline VPA in Model 1, these individuals may have had a restricted capacity for VPA to decline over follow-up. Even so, consistent with others studies in adolescents [36–39], we found no significant associations in Model 1 or 2 between adiposity and change in total PA volume or ΔMVPA (or indeed its component parts—ΔMPA and ΔVPA).

Higher levels of PA, ST and SLP at baseline were associated with greater declines in these variables over time, a phenomenon that has been described elsewhere [1, 71]. From a public health perspective this may indicate that a dual approach to PA promotion is desirable: 1) While low active youth in society must be targeted to increase levels of health-enhancing PA, 2) This should not be to the neglect of the high active quarter for whom maintenance of PA (prevention of decline) is key. That said, the magnitude of PA decline was relatively modest, and 57 % of sufficiently active participants at baseline (≥60 min MVPA/d) continued to be sufficiently active at follow-up. More worryingly, 80 % of insufficiently active participants at baseline remained so at follow-up. Further inspection of this observation revealed that 48 % of boys that failed to meet guidelines at baseline persevered to be insufficiently active at follow-up, whereas the same value was 93 % for girls. When aligned with our previous observation that 15y old girls are 3-4 times more likely to be classified as insufficiently active than boys [14], the data reaffirm that schemes to increase PA levels should primarily be directed toward girls.

This study’s strengths include the use of a pooled estimation method for body composition [47] and combined sensing estimates of habitual PA, ST and SLP. On average, participants wore the activity monitor for 105 ± 11.0 h at both time points (equivalent to >4 uninterrupted days of measurement) thereby reducing to some extent the potential impact of regression to the mean [57]. The small and homogenous sample (which restricted our capacity to investigate other determinants of change) and lack of repeat individual calibration at follow-up are limitations, but the high-comparability of participants with (*n* = 144) and without (*n* = 592) repeated activity data suggests that our sample was not biased.

## Conclusion

This study shows that intensity and gender-specific changes in PA occur from mid-to-late adolescence, and that changes in LPA and ST (but not PAEE, MPA or VPA) seem to be influenced by baseline body composition. We also observed that the greatest declines in PA occurred in those with high initial values. This may suggest that public health efforts should primarily be aimed at preventing this decline, but perhaps of greater concern is that most low-active individuals failed to increase their PA level. Contrasting the published self-reported data, our objective estimates indicate that SLP increases slightly from 15 to 17.5y of age in UK youth.

## Abbreviations

FFMI: Fat-free mass index
FMI: Fat mass index
LPA: Light physical activity
METs: Metabolic equivalents of task
MPA: Moderate physical activity
MVPA: Moderate-to-vigorous physical activity
PA: Physical activity
SLP: Sleep duration
ST: Sedentary time
PAEE: Physical activity energy expenditure
SES: Socio-economic status
VPA: Vigorous physical activity
WC: Waist circumference

## References

[1] Dumith SC, Gigante DP, Domingues MR, Kohl HW (2011). Physical activity change during adolescence: a systematic review and a pooled analysis. Int J Epidemiol.

[2] Tanaka C, Reilly JJ, Huang WY (2014). Longitudinal changes in objectively measured sedentary behaviour and their relationship with adiposity in children and adolescents: systematic review and evidence appraisal. Obes Rev.

[3] Wolfson AR, Carskadon MA (1998). Sleep schedules and daytime functioning in adolescents. Child Dev.

[4] Lytle LA, Murray DM, Laska MN, Pasch KE, Anderson SE, Farbakhsh K (2013). Examining the longitudinal relationship between change in sleep and obesity risk in adolescents. Health Educ Behav.

[5] Mitchell JA, Rodriguez D, Schmitz KH, Audrain-McGovern J (2013). Sleep duration and adolescent obesity. Pediatrics.

[6] Spruyt K, Gozal D (2011). Pediatric sleep questionnaires as diagnostic or epidemiological tools: a review of currently available instruments. Sleep Med Rev.

[7] Bauer KM, Blunden S (2008). How accurate is subjective reporting of childhood sleep patterns? A review of the literature and implications for practice. Curr Pediatr Rev.

[8] Lauderdale DS, Knutson KL, Yan LL, Liu K, Rathouz PJ (2008). Self-reported and measured sleep duration How similar Are they?. Epidemiology.

[9] Knutson KL, Lauderdale DS (2007). Sleep duration and overweight in adolescents: self-reported sleep hours versus time diaries. Pediatrics.

[10] De Vries SI, Van Hirtum HW, Bakker I, Hopman-Rock M, Hirasing RA, Van Mechelen W (2009). Validity and reproducibility of motion sensors in youth: a systematic update. Med Sci Sports Exerc.

[11] Lubans DR, Hesketh K, Cliff DP, Barnett LM, Salmon J, Dollman J (2011). A systematic review of the validity and reliability of sedentary behaviour measures used with children and adolescents. Obes Rev.

[12] Nader PR, Bradley RH, Houts RM, McRitchie SL, O’Brien M (2008). Moderate-to-vigorous physical activity from ages 9 to 15 years. JAMA.

[13] Department of Health, Physical Activity, Health Improvement and Protection (2011). Start active, stay active: a report on physical activity from the four home countries’ chief medical officers. Start active, stay active: a report on physical activity from the four home countries’ chief medical officers.

[14] Collings PJ, Wijndaele K, Corder K, Westgate K, Ridgway CL, Dunn V (2014). Levels and patterns of objectively-measured physical activity volume and intensity distribution in UK adolescents: the ROOTS study. Int J Behav Nutr Phys Act.

[15] Butte NF, Puyau MR, Adolph AL, Vohra FA, Zakeri I (2007). Physical activity in nonoverweight and overweight Hispanic children and adolescents. Med Sci Sports Exerc.

[16] Treuth MS, Hou N, Young DR, Maynard LM (2005). Accelerometry-measured activity or sedentary time and overweight in rural boys and girls. Obes Res.

[17] Kwon S, Janz KF, Burns TL, Levy SM (2011). Association between light-intensity physical activity and adiposity in childhood. Pediatr Exerc Sci.

[18] Carson V, Ridgers ND, Howard BJ, Winkler EA, Healy GN, Owen N (2013). Light-intensity physical activity and cardio metabolic biomarkers in US adolescents. PLoS One.

[19] Pate RR, O’Neill JR, Lobelo F (2008). The evolving definition of “sedentary”. Exerc Sport Sci Rev.

[20] Ekelund U, Hildebrand M, Collings PJ (2014). Physical activity, sedentary time and adiposity during the first two decades of life. Proc Nutr Soc.

[21] Tremblay MS, LeBlanc AG, Kho ME, Saunders TJ, Larouche R, Colley RC (2011). Systematic review of sedentary behaviour and health indicators in school-aged children and youth. Int J Behav Nutr Phys Act.

[22] Beebe DW (2011). Cognitive, behavioral, and functional consequences of inadequate sleep in children and adolescents. Pediatr Clin North Am.

[23] Shochat T, Cohen-Zion M, Tzischinsky O (2014). Functional consequences of inadequate sleep in adolescents: A systematic review. Sleep Med Rev.

[24] Matricciani LA, Olds TS, Blunden S, Rigney G, Williams MT (2012). Never enough sleep: a brief history of sleep recommendations for children. Pediatrics.

[25] Collings PJ, Wijndaele K, Corder K, Westgate K, Ridgway CL, Sharp SJ, Atkin AJ, Bamber D, Goodyer I, Brage S, Ekelund U: Prospective associations between sedentary time, sleep duration and adiposity in adolescents. Sleep Medicine 2015; doi:10.1016/j.sleep.2015.02.532.. 10.1016/j.sleep.2015.02.532 PMC4465960 25959093

[26] Spruyt K, Molfese DL, Gozal D (2011). Sleep duration, sleep regularity, body weight, and metabolic homeostasis in school-aged children. Pediatrics.

[27] Kwon S, Janz KF, Burns TL, Levy SM (2011). Effects of adiposity on physical activity in childhood: Iowa bone development study. Med Sci Sports Exerc.

[28] Metcalf BS, Hosking J, Jeffery AN, Voss LD, Henley W, Wilkin TJ (2011). Fatness leads to inactivity, but inactivity does not lead to fatness: a longitudinal study in children (EarlyBird 45). Arch Dis Child.

[29] Burgi F, Meyer U, Granacher U, Schindler C, Marques-Vidal P, Kriemler S (2011). Relationship of physical activity with motor skills, aerobic fitness and body fat in preschool children: a cross-sectional and longitudinal study (Ballabeina). Int J Obes.

[30] Altenburg TM, Singh AS, Van Mechelen W, Brug J, Chinapaw MJ (2012). Direction of the association between body fatness and self-reported screen time in Dutch adolescents. Int J Behav Nutr Phys Act.

[31] Hjorth MF, Chaput JP, Ritz C, Dalskov SM, Andersen R, Astrup A (2014). Fatness predicts decreased physical activity and increased sedentary time, but not vice versa: support from a longitudinal study in 8- to 11-year-old children. Int J Obes.

[32] Basterfield L, Adamson AJ, Frary JK, Parkinson KN, Pearce MS, Reilly JJ (2011). Longitudinal study of physical activity and sedentary behavior in children. Pediatrics.

[33] Ekelund U, Brage S, Besson H, Sharp S, Wareham NJ (2008). Time spent being sedentary and weight gain in healthy adults: reverse or bidirectional causality?. Am J Clin Nutr.

[34] Hasler G, Buysse DJ, Klaghofer R, Gamma A, Ajdacic V, Eich D (2004). The association between short sleep duration and obesity in young adults: A 13-year prospective study. Sleep.

[35] Guidolin M, Gradisar M (2012). Is shortened sleep duration a risk factor for overweight and obesity during adolescence? A review of the empirical literature. Sleep Med.

[36] Hallal PC, Reichert FF, Ekelund U, Dumith SC, Menezes AM, Victora CG (2012). Bidirectional cross-sectional and prospective associations between physical activity and body composition in adolescence: birth cohort study. J Sports Sci.

[37] Ekelund U, Luan J, Sherar LB, Esliger DW, Griew P, Cooper A (2012). Moderate to vigorous physical activity and sedentary time and cardio metabolic risk factors in children and adolescents. JAMA.

[38] Knowles AM, Niven AG, Fawkner SG, Henretty JM (2009). A longitudinal examination of the influence of maturation on physical self-perceptions and the relationship with physical activity in early adolescent girls. J Adolescence.

[39] Cumming SP, Sherar LB, Esliger DW, Riddoch CJ, Malina RM (2014). Concurrent and prospective associations among biological maturation, and physical activity at 11 and 13 years of age. Scand J Med Sci Sports.

[40] Finne E, Bucksch J, Lampert T, Kolip P (2011). Age, puberty, body dissatisfaction, and physical activity decline in adolescents. Results of the German Health Interview and Examination Survey (KiGGS). Int J Behav Nutr Phys Act.

[41] Telama R, Yang X, Leskinen E, Kankaanpaa A, Hirvensalo M, Tammelin T (2014). Tracking of physical activity from early childhood through youth into adulthood. Med Sci Sports Exerc.

[42] Goodyer IM, Croudace T, Dunn V, Herbert J, Jones PB (2010). Cohort profile: risk patterns and processes for psychopathology emerging during adolescence: the ROOTS project. Int J Epidemiol.

[43] Brage S, Ekelund U, Brage N, Hennings MA, Froberg K, Franks PW (2007). Hierarchy of individual calibration levels for heart rate and accelerometry to measure physical activity. J Appl Physiol.

[44] Brage S, Brage N, Franks PW, Ekelund U, Wong MY, Andersen LB (2004). Branched equation modeling of simultaneous accelerometry and heart rate monitoring improves estimate of directly measured physical activity energy expenditure. J Appl Physiol.

[45] Wolfson AR, Carskadon MA, Acebo C, Seifer R, Fallone G, Labyak SE (2003). Evidence for the validity of a sleep habits survey for adolescents. Sleep.

[46] Brage S, Westgate K, Wijndaele K, Godinho J, Griffin S, Wareham N: Evaluation of a method for minimising diurnal information bias in objective sensor data. Int Conf Amb Mon Phys Act Mov 2013.

[47] Wells JC, Williams JE, Haroun D, Fewtrell MS, Colantuoni A, Siervo M (2009). Aggregate predictions improve accuracy when calculating metabolic variables used to guide treatment. Am J Clin Nutr.

[48] Mellits ED, Cheek DB (1970). The assessment of body water and fatness from infancy to adulthood. Monogr Soc Res Child Dev.

[49] Morgenstern BZ, Mahoney DW, Warady BA (2002). Estimating total body water in children on the basis of height and weight: a reevaluation of the formulas of Mellits and Cheek. J Am Soc Nephrol.

[50] Chumlea WC, Schubert CM, Reo NV, Sun SS, Siervogel RM (2005). Total body water volume for white children and adolescents and anthropometric prediction equations: the Fels Longitudinal Study. Kidney Int.

[51] Deurenberg P, Weststrate JA, Seidell JC (1991). Body-mass index as a measure of body fatness-Age-specific and Sex-specific prediction formulas. Br J Nutr.

[52] Pietrobelli A, Faith MS, Allison DB, Gallagher D, Chiumello G, Heymsfield SB (1998). Body mass index as a measure of adiposity among children and adolescents: a validation study. J Pediatr.

[53] Foster BJ, Platt RW, Zemel BS (2012). Development and validation of a predictive equation for lean body mass in children and adolescents. Ann Hum Biol.

[54] Haroun D, Taylor SJC, Viner RM, Hayward RS, Darch TS, Eaton S (2010). Validation of bioelectrical impedance analysis in adolescents across different ethnic groups. Obesity.

[55] Cole TJ, Freeman JV, Preece MA (1995). Body mass index reference curves for the UK, 1990. Arch Dis Child.

[56] McCarthy HD, Jarrett KV, Crawley HF (2001). The development of waist circumference percentiles in British children aged 5.0-16.9 y. Eur J Clin Nutr.

[57] Barnett AG, van der Pols JC, Dobson AJ (2005). Regression to the mean: what it is and how to deal with it. Int J Epidemiol.

[58] Health Survey for England 2008: Physical activity and fitness [http://www.hscic.gov.uk/pubs/hse08physicalactivity (accessed May 29, 2013)]

[59] Corder K, Sharp SJ, Atkin AJ, Griffin SJ, Jones AP, Ekelund U, van Sluijs EM: Change in objectively measured physical activity during the transition to adolescence. Br J Sports Med 2013. doi:10.1136/bjsports-2013-093190. 10.1136/bjsports-2013-093190 PMC4453714 24273308

[60] Freedson P, Pober D, Janz KF (2005). Calibration of accelerometer output for children. Med Sci Sports Exerc.

[61] Collings PJ, Brage S, Ridgway CL, Harvey NC, Godfrey KM, Inskip HM (2013). Physical activity intensity, sedentary time, and body composition in preschoolers. Am J Clin Nutr.

[62] Janssen I, Leblanc AG (2010). Systematic review of the health benefits of physical activity and fitness in school-aged children and youth. Int J Behav Nutr Phys Act.

[63] Ohayon MM, Carskadon MA, Guilleminault C, Vitiello MV (2004). Meta-analysis of quantitative sleep parameters from childhood to old age in healthy individuals: developing normative sleep values across the human lifespan. Sleep.

[64] Collishaw S, Maughan B, Natarajan L, Pickles A (2010). Trends in adolescent emotional problems in England: a comparison of two national cohorts twenty years apart. J Child Psychol Psychiatry.

[65] Corder K, Van Sluijs EM, Ekelund U, Jones AP, Griffin SJ (2010). Changes in children’s physical activity over 12 months: longitudinal results from the SPEEDY study. Pediatrics.

[66] Hutcheon JA, Chiolero A, Hanley JA (2010). Random measurement error and regression dilution bias. BMJ.

[67] Van Breukelen GJP (2006). ANCOVA versus change from baseline had more power in randomized studies and more bias in nonrandomized studies. J Clin Epidemiol.

[68] Fitzmaurice G (2001). A conundrum in the analysis of change. Nutrition.

[69] Glymour MM, Weuve J, Berkman LF, Kawachi I, Robins JM (2005). When is baseline adjustment useful in analyses of change? An example with education and cognitive change. Am J Epidemiol.

[70] Dugravot A, Gueguen A, Kivimaki M, Vahtera J, Shipley M, Marmot MG (2009). Socioeconomic position and cognitive decline using data from two waves: what is the role of the wave 1 cognitive measure?. J Epidemiol Commun H.

[71] Corder K, Craggs C, Jones AP, Ekelund U, Griffin SJ, Van Sluijs EM (2013). Predictors of change differ for moderate and vigorous intensity physical activity and for weekdays and weekends: a longitudinal analysis. Int J Behav Nutr Phys Act.

